# Early viability assessment of a Business-to-Consumer (B2C) model for digital diabetes screening in Switzerland

**DOI:** 10.1186/s12913-026-14075-3

**Published:** 2026-01-28

**Authors:** Wasu Mekniran, Tobias Kowatsch

**Affiliations:** 1 Department of Management, Technology and Economics, ETH Zürich, Zürich, Switzerland; 2 Institute of Technology Management, University of St. Gallen, St. Gallen, Switzerland; 3 School of Medicine, University of St. Gallen, St. Gallen, Switzerland; 4 Institute for Implementation Science in Health Care, University of Zürich, Zürich, Switzerland

**Keywords:** Business model, Breakeven analysis, Venture valuation, Digital biomarkers, Diabetes screening, Prevention

## Abstract

**Background:**

Type 2 diabetes (T2D) represents a rapidly growing public health and economic burden. Although early intervention can reverse the progression of prediabetes, traditional risk screening remains underutilized. Digital biomarkers derived from smartphones and wearables offer scalable real-time detection, yet financial barriers constrain their integration into health systems. This study assesses the viability of a Business-to-Consumer (B2C) digital diabetes screening venture within the Swiss healthcare system.

**Methods:**

The Innovating in Healthcare Framework was applied to evaluate system alignment across six factors: structure, financing, public policy, technology, consumers, and accountability. Financial viability was modeled using a Monte Carlo program for probabilistic breakeven estimation, and one- and two-way sensitivity analyses for key funnel, price, and CAC variables. A discounted cash flow model assessed value creation. Sustainability was evaluated across four dimensions: revenue potential, cost efficiency, managerial scalability, and technological adaptability.

**Results:**

The venture showed strong alignment with consumer readiness, technology, and accountability, but weak fit in financing, public policy, and system structure. Financial modeling indicated positive cash flow in Year 4, with a 57% probability of breakeven within seven years. At 2917 users in Year 7, cumulative cash flow was slightly below zero. Profitability becomes feasible only when price ≥CHF 40, CAC ≤CHF 200, and screening participation ≥10%. Against a-priori thresholds, breakeven probability and NPV remain insufficient, while IRR often exceeds 20%. Long-term viability requires these conditions or transitioning to reimbursed B2B pathways.

**Conclusions:**

A B2C model can reach financial viability under favorable price and acquisition thresholds, but its long-term sustainability ultimately depends on regulatory validation that enables reimbursement.

**Supplementary information:**

The online version contains supplementary material available at 10.1186/s12913-026-14075-3.

## Background

Type 2 diabetes (T2D) presents a major global health and economic challenge, with projections indicating it will affect 643 million people worldwide by 2030 (International Diabetes Federation, 2025). This condition typically progresses through a clinically silent prediabetic phase of insulin resistance, during which early screening and intervention can enable reversal [1]. Switzerland’s healthcare system, known for high per-capita spending, universal mandatory insurance, and quality of care, paradoxically struggles with widespread prevention [2–5]. Although prevention is declared a policy goal, the prevailing fee-for-service incentives continue to prioritize acute treatment over proactive preventive measures [6]. Landmark trials, such as the Diabetes Prevention Program (DPP), have conclusively shown that structured lifestyle interventions can reduce diabetes risk by up to 58%, substantially outperforming pharmacological approaches such as metformin [7–9]. This evidence has prompted initiatives worldwide promoting behavioral coaching, dietary counseling, and physical activity [7, 10–13]. However, these conventional programs often face limitations in timely detection and long-term scalability of such screening campaigns [2]. Furthermore, opportunistic and symptom-based in-clinic screening models severely restrict reach and detection rate, particularly among asymptomatic prediabetic individuals [14, 15]. In Switzerland, the prevalence of undiagnosed prediabetes is estimated at 11–30% among adults and up to 80% among older adults [2, 16], a critical oversight that contributes to escalating delays in preventing complications such as cardiovascular disease, nephropathy, and stroke [2, 17]. Traditional outreach campaigns have also proven inefficient: a pharmacy-based campaign in the Swiss canton of Vaud involving 190 pharmacies cost CHF 330,600 for two weeks, reached fewer than 1% of the target group (*n* = 4,222), while identified 30.4% as prediabetic [2]. This “Prevention gap” underscores the pressing need for accessible and scalable screening modalities. In Switzerland, reimbursement is limited mainly to cancer screenings, while other chronic condition screenings, including diabetes and hypertension, are not covered until diagnosis is established in standard care, requiring alternative financing for preventive screening providers [18, 19].

Digital Health Technologies (DHT), which include mobile applications and wearables, offer scalable tools to monitor health and support lifestyle interventions (FDA, 2020). Recent advances in AI-driven digital biomarkers for T2D enable the continuous analysis of user-generated data, transforming everyday physiological signals into actionable risk assessments [20]. Such biomarkers can be derived from electrocardiogram (ECG), photoplethysmography (PPG), accelerometry (ACC), electrodermal activity (EDA), and skin temperature (SKT), providing personalized feedback while potentially reducing healthcare provider burden [20–24]. Evidence from the United States and the United Kingdom shows that digitally enhanced diabetes prevention programs are cost-effective, but their impact depends critically on early detection and sustained user engagement [25–29].

**Fig. 1:**
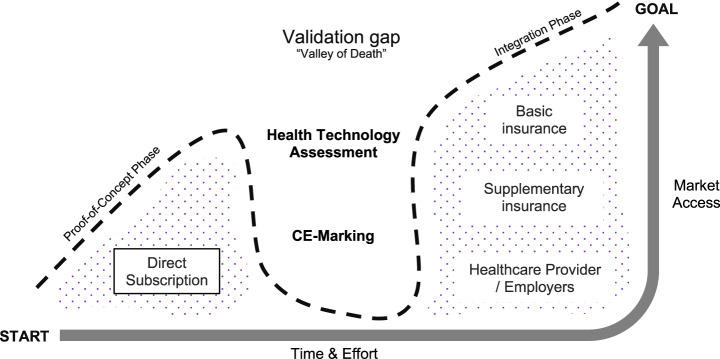
Market access pathway for digital diabetes screening in Switzerland, illustrating the progression from proof-of-concept through B2C entry to potential integration into insurer reimbursement and statutory care

In Switzerland, digital adoption is high, with 97% of adults being online, 90% owning smartphones, and 51% tracking their physical activity (28% of whom do so daily) [30, 31]. Yet, conversion from casual tracking to structured digital prevention remains low, creating uncertainty for a Business-to-Consumer (B2C) model. Only 40% of adults demonstrate above-basic digital literacy [31], and privacy concerns remain acute among older and lower-income groups, which directly affect customer acquisition costs, churn rates, and ultimately willingness-to-pay. This paradox underscores that while technical capacity exists, adoption at scale necessitates robust implementation strategies, such as simplified subscription pricing and physician endorsements, rather than relying solely on consumer digital literacy [32, 33]. The absence of a dedicated reimbursement pathway for digital health and the lack of tailored Health Technology Assessment (HTA) for digital interventions create a fundamental business model gap (Research2Guidance, 2024). While international programs, such as Germany’s DiGA (FIDMD, 2020) or the US FDA Pre-Certification Pilot, provide routes to reimbursement (Van Norman, 2016), digital diabetes screening in Switzerland must initially rely on an interim pathway that allows ventures to generate real-world evidence for safety and performance compliances, and test demand, as shown in Fig. 1. Still, its financial viability and long-term sustainability of B2C model remain largely unexplored. Previous experiences with global platforms like Omada Health and Livongo demonstrate both the necessity and risks of overreliance on short-term B2C revenue without validated outcomes [34].

This study addresses this validation gap by assessing the viability of a hypothetical B2C digital screening venture in Switzerland and answering three research questions: (RQ1) To what extent does a B2C digital diabetes screening venture align with the Swiss healthcare system? (RQ2) What financial thresholds must be reached to achieve breakeven and investment viability during the B2C phase? (RQ3) How is the implementation sustainability of this B2C model in Switzerland, particularly as it seeks to bridge towards B2B integration?

## Methods

### Study design

We examine system alignment (RQ1), financial thresholds for breakeven and investment viability (RQ2), and sustainability with pathways to B2B integration (RQ3). An overview of research questions and methods is provided in Table 1.

**Table 1:** Overview of the study design, aligning each research question (RQ1–RQ3) with data sources and analytical methods

Research Question	Data Collection	Data Analysis
(RQ1) To what extent does a B2C digital diabetes screening venture align with the Swiss healthcare system?	- Targeted, non-systematic literature review of academic and gray sources	- Qualitative description using Herzlinger’s Six Factors of Innovation - Scoring Likert-scale [1–4] by three coauthors - Consensus for scoring reconciliation
(RQ2) What financial thresholds must be reached to achieve breakeven and investment viability during the B2C phase?	- Input assumptions based on RQ1 on financial metrics, i.e., Swiss pricing, market volumes, and payer policies.	- Breakeven probability analysis with pro forma model - Willingness-to-pay estimation - Cash flow valuation with discount rates
(RQ3) How is the implementation sustainability of this B2C model in Switzerland, particularly as it seeks to bridge towards B2B integration?	- Synthesis of empirical insights from RQ1 and RQ2	- Descriptive synthesis across four dimensions: revenue potential, cost efficiency, management scalability, and technological adaptability

### Evidence from literature

A targeted non-systematic literature review informed the parameterization of RQ1 (system alignment) and RQ2 (financial modeling). Similar to prior work [35], a non-systematic approach was deliberately chosen because the analytic goal was not to synthesize clinical effectiveness evidence, but to assemble decision-grade quantitative inputs across heterogeneous domains: epidemiology, diagnostic accuracy, adoption funnels, willingness-to-pay, comparator costs, and regulatory requirements, where relevant public data are distributed across peer-reviewed publications, gray literature, market reports, and commercial pricing lists.

The searches were conducted in PubMed, Web of Science, and Google Scholar (2010–2025), using keywords such as digital diabetes prevention, digital biomarkers, healthcare business, and Switzerland. Inclusion emphasized empirical data on B2C digital health models, cost benchmarks, adoption patterns, and the Swiss regulatory context. Supplementary evidence was provided by the World Health Organization, International Diabetes Federation, Swiss Federal Statistical Office, and market research (e.g., RockHealth, Research2Guidance), as well as company reports from Omada Health, Livongo, and Oviva. Key financial inputs are summarized in Table 2. Inclusion prioritized empirical or quantitative sources that provided parameters relevant to the six evidence domains (Epidemiology, clinical performance, adoption, pricing, cost comparators, compliance); purely qualitative studies or sources without extractable estimates were excluded. To maintain transparency, all extracted parameters, their sources, and ranges are reported in Appendix A. We did not conduct a formal study-level risk-of-bias assessment. Instead, evidence credibility was evaluated by source type (peer-reviewed publications, registry data, governmental statistics, or market reports) and by triangulating estimates across independent evidence categories where feasible (e.g., government registries and market reports). This approach aligns with established methods in development-focused Health Technology Assessment (HTA) and business-model evaluation, which prioritize transparency of financial assumptions over exhaustive literature retrieval [35–39].

**Table 2:** Parameter inputs for the pro forma financial model for a B2C digital diabetes screening venture in Switzerland

Input Assumptions	Median	Min	Max	Rationale
Adult Population	6,500,000	-	-	Swiss national adult (21+) population
Screening Eligibility	40%	30%	50%	USPSTF-aligned eligibility criteria
Impaired Fasting Glucose Population	11%	10%	12%	IDF-based IFG prevalence range
Undiagnosed Population	30%	11%	80%	Swiss underdiagnosis estimates
Screening Participation	10%	5%	15%	Observed screening participation constraints
Adoption Rate	1.5%	0.5%	2.0%	Digital health uptake funnel benchmarks
Paid Conversion Rate	40%	20%	60%	Conversion expectations
Churn Rate (Annual)	55%	40%	70%	Digital churn parameters
Customer Lifetime (Years)	1.5	1.0	2.0	Lifetime derived from annual subscription plan
Customer Acquisition Cost per User	220	150	350	CAC benchmarks for B2C channel
Subscription Price per Month	40	20	60	Swiss WTP comparator anchors
Technician per User Ratio	700	500	900	Coaching workload norms
Manager per Technician Ratio	15	10	20	Lean-operations supervision ratio
Personnel Costs (Technicians)	95,000	85,000	110,000	Swiss software developer salary
Personnel Costs (Managers)	120,000	100,000	140,000	Swiss management salary
Office Supplies per Worker	1,200	800	1,500	SME office expenditures
Office Rent per Worker	10,000	8,000	12,000	Commercial rent
Call-center per User	20	15	30	Customer support outsourcing costs
Backend Services per User	35	25	45	Cloud-service cost benchmarks
App Development	300,000	250,000	400,000	Standard mobile-app build range
App Maintenance (Annual)	50,000	40,000	60,000	Annual SaaS upkeep norms
CE MDR Certification Cost	20,000	10,000	30,000	MDR Rule 11 CE IIa certification
revFADP Compliance Cost	25,000	15,000	40,000	Swiss data-protection annual costs
Discount Rate	18%	12%	30%	VC risk-adjusted return range

### Herzlinger’s six factors of innovation analysis

For RQ1, system alignment was assessed using Herzlinger’s Six Factors of Innovation; this served as a diagnostic heuristic for early system alignment, not as a psychometric tool. We used the six-factor healthcare innovation framework because it provides a comprehensive, decision-oriented structure to evaluate consumer-facing health innovations. Unlike case-based templates, the framework explicitly incorporates structural, financial, policy, technological, consumer, and accountability elements needed for early-stage venture assessment.

For each of six factors, the assessment involved specific data sources. For ‘Structure’, this included an analysis of Switzerland’s decentralized cantonal electronic health record (EHR) systems and the landscape of established digital health platforms and insurer wellness programs. For ‘Financing’, the assessment drew upon market comparables for Swiss preventive services and public disclosures from leading digital health companies to inform willingness-to-pay estimation and input parameters for the discounted cash flow (DCF) model. ‘Public Policy’ and ‘Accountability’ involved analyzing existing Swiss regulatory frameworks, HTA methodologies, and discussions regarding reimbursement pathways for digital health technologies. For ‘Technology’, the assessment considers data on Swiss internet penetration, wearable adoption rates, and the technical feasibility of biometric data integration. For ‘Consumers’, insights were gathered from the digitalswitzerland survey (2025) on digital literacy and health app usage, alongside literature on user engagement in digital health interventions. In the Discussion, we compare this approach to alternative models (e.g., NPV decomposition, evolutionary entrepreneurship frameworks).

Three evaluators (WM, TK, MJ) independently scored each factor on a four-point Likert scale (1 = non-existent, 2 = poor, 3 = good, 4 = excellent), guided by structured questions adapted from the framework. Scoring was informed by Swiss market data, complemented by literature, and subsequently reconciled through consensus. We calculated Fleiss’ Kappa as a descriptive index of agreement; given the small number of items and conceptual breadth, Kappa was not used as a decisive psychometric statistic but was reported alongside a consensus process and independent rating. Because the analysis relied on secondary data and expert interpretation, the convergence of literature, empirical evidence, and six-factor rating was used to minimize bias and strengthen trustworthiness (Chang, 1994).

To operationalize the business model assessment, we rendered insights from Herzlinger’s factors into quantifiable inputs for a profit-and-loss (P&L) simulation. Specifically, Structure informed market size and penetration; Financing defined pricing, payer coverage, and discount rates; Consumers determined adoption, churn, and subscription duration; Public Policy specified compliance costs and reimbursement delays; Technology captured R&D, scalability, and marginal costs; and Accountability reflected validation costs and adoption delays. This mapping linked qualitative system-fit insights directly to financial assumptions, grounding the P&L model in both theory and Swiss market realities. Comparator pathways were considered only to the extent that opportunistic screening provided a willingness-to-pay benchmark, while regulatory costs were explicitly included and reflected in the financial model, without undertaking a full HTA or payer budget-impact analysis.

### Financial modeling

For RQ2, a seven-year pro forma P&L model was constructed using the Monte Carlo method with 5000 iterations to simulate retention-adjusted revenues, operational costs, regulatory obligations, and net cash flow for a subscription-based B2C venture. Breakeven analysis probability is identified each year in which cumulative revenues equal total costs, indicating the minimum adoption-retention threshold for operational viability. Sensitivity analysis examined how the adoption and churn parameters, informed by RQ1, affected the breakeven and profitability.

To estimate financial thresholds for breakeven and investment viability, we triangulated willingness-to-pay (WTP) against market benchmarks from Swiss preventive services, digital health apps, and home testing. Clinical diagnostics such as HbA1c (CHF 16) and OGTT (CHF 30–40) are relatively inexpensive [40] but require clinic visits and lack follow-up. By contrast, home tests cost CHF 70–75 [40, 41], while digital coaching sessions such as Oviva are priced at CHF 40 per 15 minutes [42]. These benchmarks indicate the range of existing retail pricing in Switzerland.

To evaluate investment attractiveness, we applied a standard discounted cash flow (DCF) analysis, which estimates the present value of future cash flows under varying investor risk profiles. Financial performance was evaluated using both absolute value metrics (NPV) and rate-based metrics (IRR), acknowledging that early-stage digital health ventures may display divergent signals across these measures due to scale effects and the timing of cash flows. Terminal value was estimated using an earnings multiple of 10 to approximate long-term potential beyond the model horizon. A priori viability criteria were defined as: (i) IRR ≥ 20%, (ii) probability of breakeven by Year 5 ≥ 50%, and (iii) positive NPV under median assumptions. These thresholds were selected to reflect investor requirements for strong risk-adjusted returns and timely breakeven to compensate for high adoption uncertainty.

All variable assumptions are reported in Table 2, and financial logic and equations in Appendix A. These values represent modeled assumptions based on public benchmarks; no proprietary company data were used. The full uncertainty program, which includes Monte Carlo simulation, one-way sensitivity analysis and two-way elasticity analysis, is summarized in Appendix A, and all underlying code and reproducible scripts are available in the public repository (DOI:10.17605/OSF.IO/VH2FR). Together, these methods linked system-fit assumptions to financial projections, allowing us to test whether a B2C model could generate sufficient return to motivate early-stage founders and attract investment in Switzerland.

### Sustainability assessment

For RQ3, we evaluated the sustainability of the B2C model’s implementation across four dimensions: revenue potential, cost efficiency, managerial scalability, and technological adaptability. Revenue potential was captured by assessing whether pricing and adoption could generate sufficient recurring income, while cost efficiency reflected the extent to which fixed and variable costs declined per user as the scale increased. Managerial scalability assessed the capacity to expand operations without disproportionate increases in staffing or organizational complexity, and technological adaptability considered whether the digital infrastructure could integrate with clinical workflows and insurer platforms. This synthesis of the findings from RQ1 (system alignment) and RQ2 (financial modeling) enabled us to assess whether the B2C venture could remain viable and provide a bridge toward healthcare system integration.

## Results

### (RQ1) to what extent does a B2C digital diabetes screening venture align with the Swiss healthcare system?

#### Venture type

**Fig. 2:**
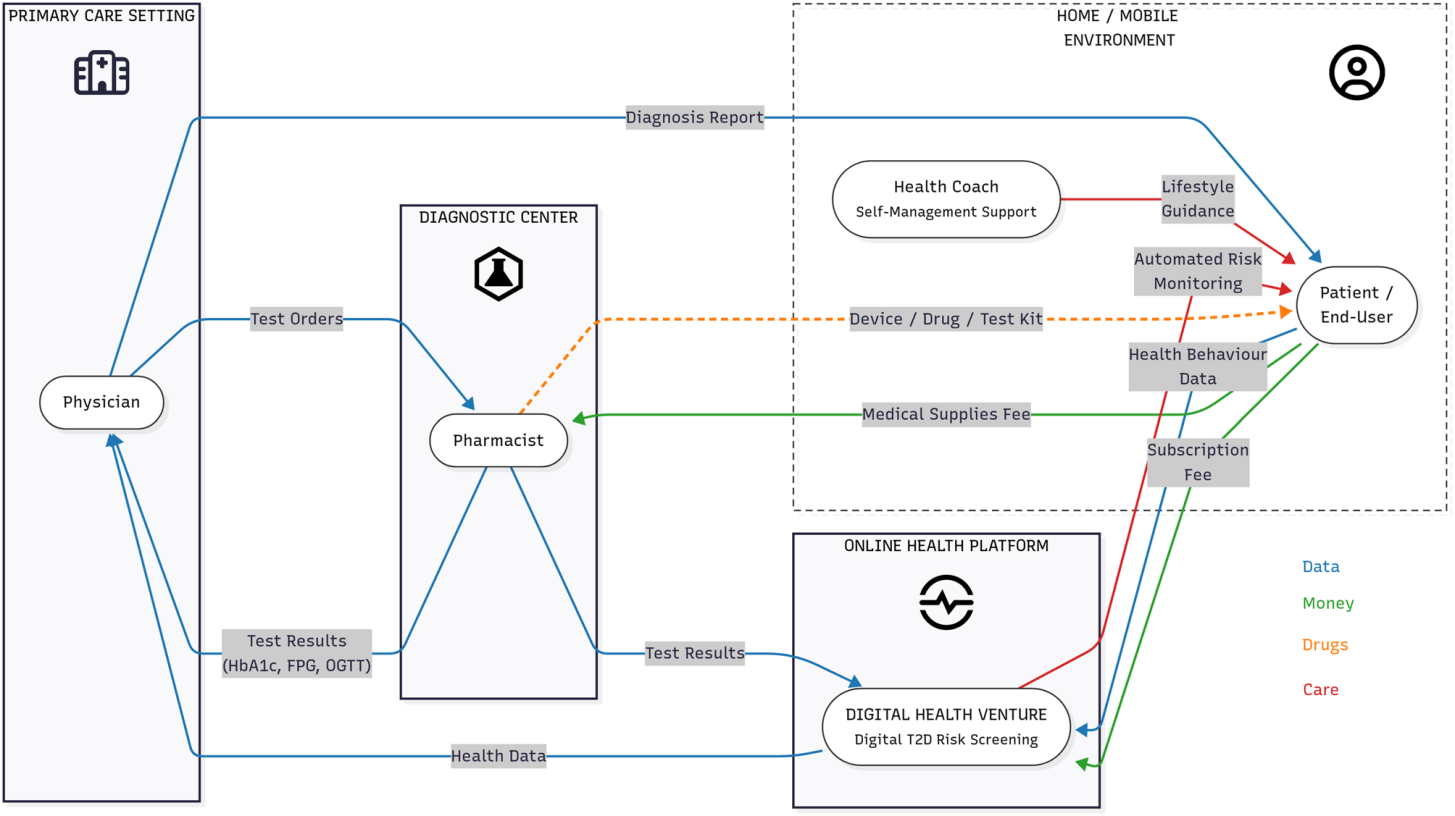
Stakeholder map of type 2 diabetes prevention, contrasting traditional opportunistic screening (left panel) with digital B2C screening models (right panel). The diagram illustrates how digital self-delivery pathways alter the relationships among patients, providers, payers, and support services

**Fig. 3:**
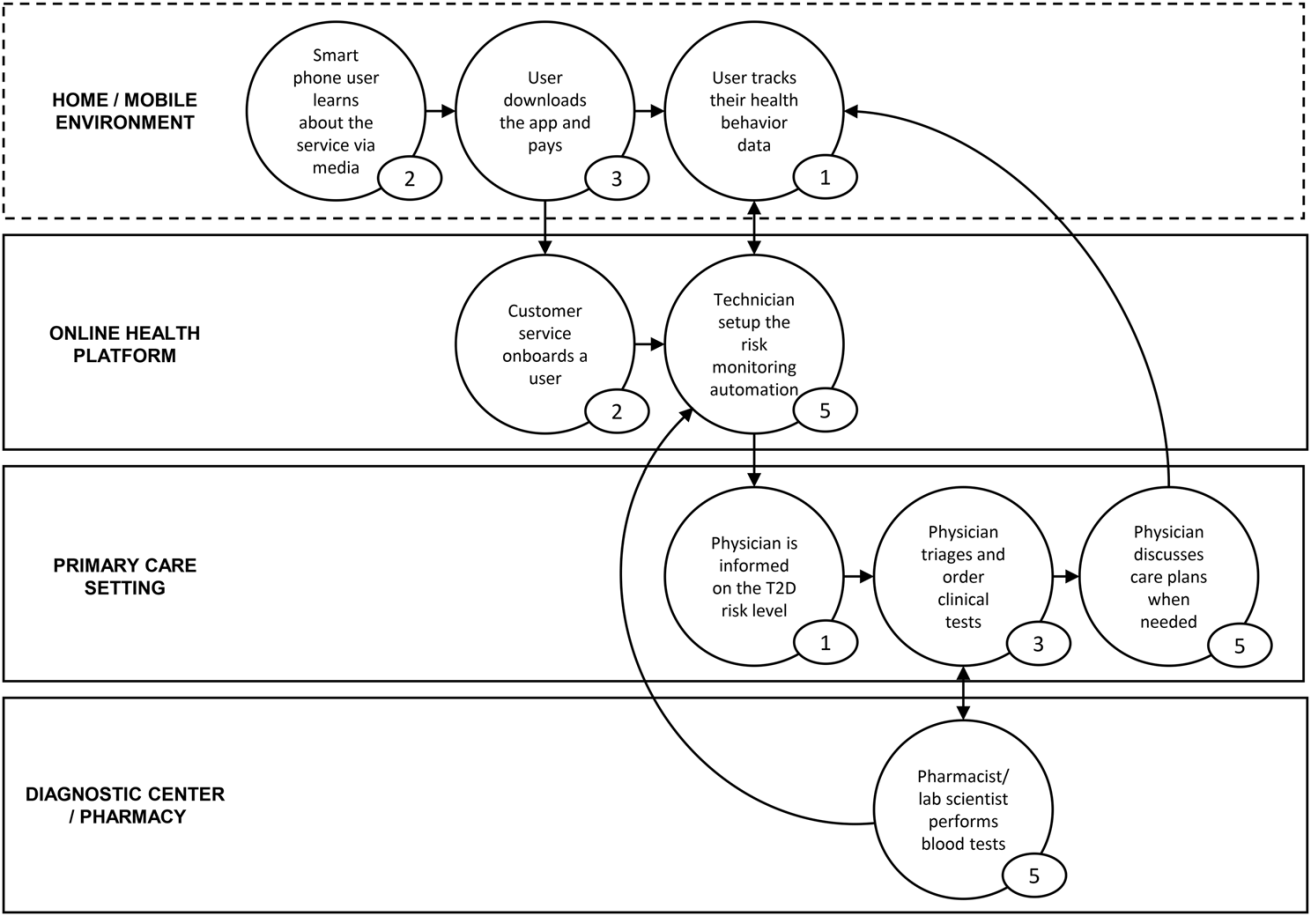
Care experience flow map of a Swiss B2C digital diabetes screening model. The map illustrates end-user interactions, time spent in each state, and exchanges between care environments. Adapted from patient shadowing the patient and family centered care [35]

To position the hypothetical B2C model within the broader prevention landscape, we compared it with three established pathways as illustrated in Fig. 2: opportunistic in-clinic screening (Left), pharmacy-based point-of-care testing (Mid), and a digital pathway (Right). These pathways differ structurally in how patients, providers, payers, and support services interact, with the B2C route bypassing traditional gatekeepers and enabling consumer engagement in the home environment. Figure 3 further visualizes these differences by mapping end-user actions, time in each state, and cross-environment exchanges within the B2C flow, making explicit how digital self-delivery reshapes both operational burden and prevention reach.

##### Alignment with Herzlinger’s six factors of innovation

**Fig. 4:**
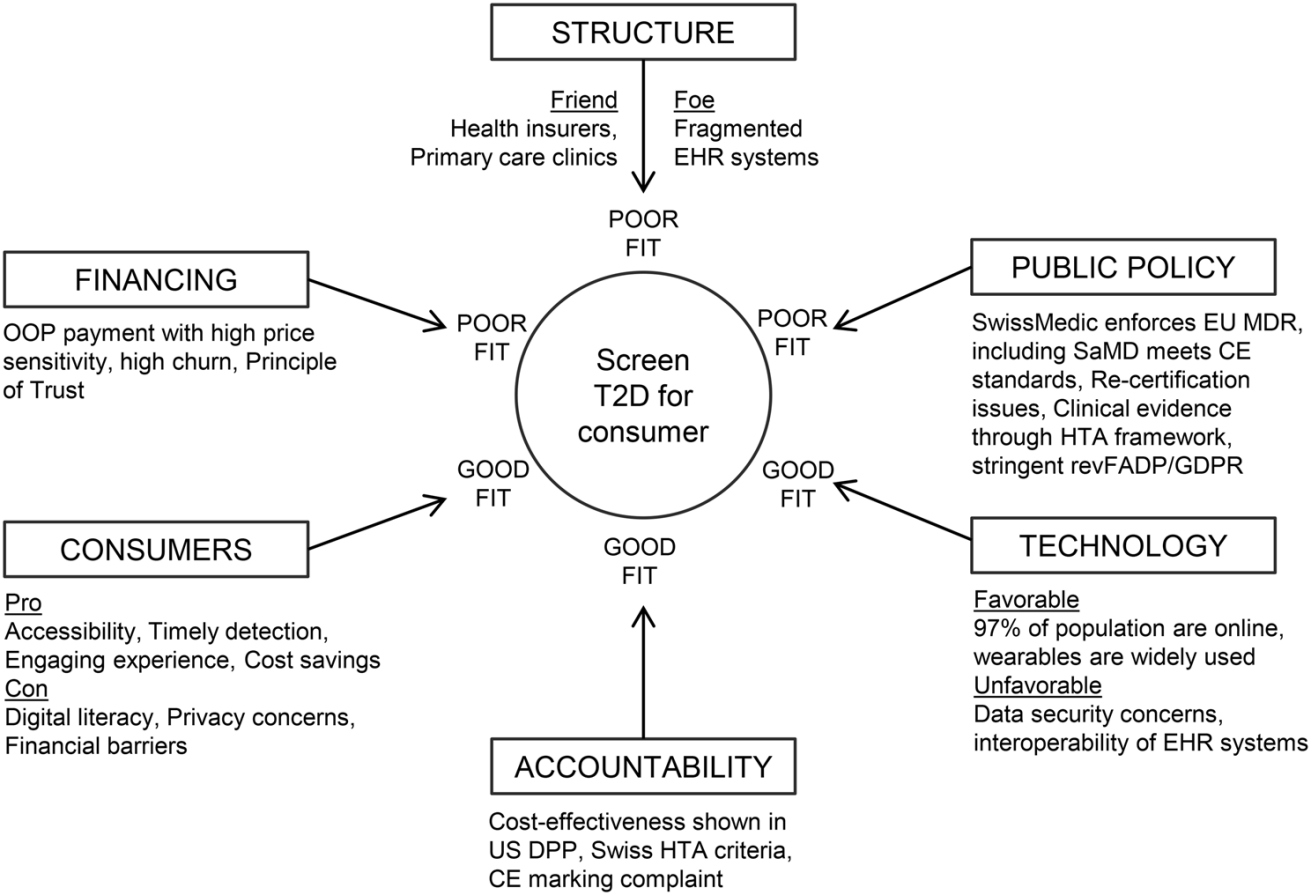
Alignment with Herzlinger’s six factors of innovation for a Swiss B2C diabetes screening venture, adapted from Herzlinger [43]

The results are presented in the following categories: structure, financing, public policy, technology, consumers, and accountability, as illustrated in Fig. 4. Raw ratings were as follows: Structure as Poor (all raters), Consumer as Poor (TK), Good (MJ), Excellent (WM), Technology as Poor (TK), Good (MJ), Excellent (WM), Financing as Poor (WM & MJ), Good (TK), Public Policy as Non-existent (WM), Poor (MJ), Good (TK), Accountability as Poor (WM), Good (TK & MJ). Fleiss’ Kappa was −0.16, indicating substantial divergence in expert judgement across innovation domains. This divergence is interpreted as reflecting underlying system ambiguity rather than rater inconsistency. Thus, we treat the six-factor scores as a diagnostic qualitative lens, intentionally complemented by transparent quantitative modeling, rather than as a psychometric measurement scale. Hence, a structured consensus process was applied to resolve discrepancies and consolidate the assessment.

##### Structure: “Poor”

Structural alignment for a B2C diabetes screening venture in Switzerland is poor. On the positive side, insurers are legally mandated to promote prevention [19] and already operate wellness platforms such as CSS active365 and Helsana+, offering potential pathways for B2B integration [44]. Provider networks, including Hirslanden and Medbase, within ecosystems such as Compassana, could also embed digital screening into workflows, thus enhancing trust and eventual physician endorsement (Boni, 2020). However, major barriers limit independent B2C scaling. Established platforms such as mySugr and Dacadoo already dominate parts of the digital diabetes market [45]while Switzerland’s fragmented electronic health record infrastructure, comprising more than 50 decentralized systems, severely restricts data integration and demonstration of clinical outcomes, which are essential for reimbursement [46]. These weaknesses suggest that while initial user acquisition may be feasible, long-term scaling requires strategic partnerships and cross-ecosystem integration toward sustainability.

##### Financing: “Poor”

Financing alignment for a B2C diabetes screening venture in Switzerland is poor. Out-of-pocket models face high churn and price sensitivity, making early revenues fragile [47]. Although insurers face rising diabetes-related costs [6], and thus have incentives to invest in prevention [14], reimbursement for digital prevention remains limited. Existing pathways under the Swiss “Principle of Trust” permit coverage of digital tools only when prescribed by licensed providers [48], and insurers mainly integrate preventive apps into wellness platforms rather than reimbursable care. Long-term trends toward outcome-based contracts and value-based prevention align with digital health’s B2B potential [49, 50]. Yet payers require rigorous evidence of cost savings in medication use, complications, and hospitalizations before committing [14, 25, 51, 52]. Further friction arises from fragmented cantonal regulation and the absence of a unified DiGA-style reimbursement pathway [53].

Consequently, while capital and payer incentives for prevention exist, B2B financing is contingent on robust evidence of clinical efficacy and economic value [37, 38, 54]. Until such validation is achieved, the venture remains dependent on direct consumer payments, underscoring poor financing alignment and highlighting the need for a strong value proposition and a willingness-to-pay to sustain B2C market entry.

##### Public policy: “Poor”

Public policy alignment for B2C diabetes screening in Switzerland is poor. On the enabling side, Swissmedic’s adherence to EU MDR standards ensures CE marking for Software as a Medical Device (SaMD), which builds institutional trust among clinicians and payers for future B2B adoption [55]. Ongoing policy discussions on DiGA-like reforms further signal a gradual governmental embrace of digital therapeutics [56]. However, several barriers directly constrain the B2C model. Diabetes screening is not reimbursed [19], making consumer payments the only feasible entry option. Frequent software iterations require revalidation under CE rules, delaying time-to-market [32]. The revised Federal Data Protection Act (revFADP) introduces GDPR-like requirements for local data hosting and explicit consent, raising compliance costs [57]. Furthermore, the HTA criteria for digital prevention tools remain undefined, and the liability for AI-based risk scores is unclear, creating regulatory uncertainty [48, 58, 59].

Thus, while Switzerland actively promotes digital health innovation and clinical trials [60], the absence of tailored HTA frameworks, and clarity on liability leaves B2C diabetes screening ventures poorly aligned with current policy priorities.

##### Technology: “Good”

Technological alignment is good, as Switzerland offers a strong digital infrastructure, but faces notable integration challenges. High internet penetration and widespread wearable adoption (e.g., Apple Health, Fitbit, Garmin) provide a robust interface for biometric tracking, supporting AI algorithms with reliable specificity and sensitivity in T2D risk detection [61–65]. These devices can generate clinically relevant data (BMI, HbA1c, blood pressure, diet, sleep), and the ecosystem technically supports multi-stakeholder connectivity linking patients, providers, and insurers [66–68]. However, fragmented IT infrastructure across cantons and insurers limits interoperability, and the multicultural context requires linguistically adaptive interfaces and food databases to ensure usability. Thus, while Switzerland provides the technological prerequisites for B2C diabetes screening, evidence generation for B2B integration hinges on resolving interoperability and localization bottlenecks.

##### Consumer: “Good”

Consumer alignment is good, reflecting both strong potential and significant barriers. Digital adoption is high: 97% of Swiss adults are online, 90% own smartphones, and 51% track their health digitally [30]. Demand for preventive care is growing, particularly among older populations facing the rising burden of noncommunicable diseases [6, 69]. Engagement can be enhanced through behavioral design features such as gamified feedback and community challenges [66], as well as integration with insurer wellness apps offering incentives like reward points [70, 71].

However, barriers remain significant. Only 40% of adults demonstrate above-basic digital literacy [31], and privacy concerns are substantial among older and lower-income groups. These factors directly affect customer acquisition, churn, and willingness-to-pay. Broader behavioral challenges also persist: only 15% of Swiss adults meet fruit and vegetable intake recommendations, and obesity prevalence has doubled over three decades [72]. Finally, physician endorsement remains important to drive adoption among risk-averse or less tech-savvy users.

Overall, while Swiss consumers are digitally connected and increasingly open to prevention, sustained B2C adoption will depend on bridging literacy and trust gaps, linking incentives to behavioral change, and leveraging medical endorsement [73–75]. This underpins a favorable but conditional consumer alignment.

##### Accountability: “Good”

Accountability for a B2C diabetes screening model is assessed as good but demanding. Accountability in this context refers to the ability to demonstrate clinical validity, user safety, algorithmic transparency, and measurable outcomes expected by regulators, clinicians, payers, and users. Existing precedents, such as the US Diabetes Prevention Program and its digital equivalents, demonstrate clinically meaningful outcomes (e.g., 6.5% weight loss) that align with Swiss HTA criteria for effectiveness, appropriateness, and cost efficiency [29]. Regulatory oversight further strengthens accountability: CE marking requires continuous audits and safety surveillance, enhancing transparency [56]. These mechanisms can create trust among clinicians, payers, and policymakers.

At the same time, accountability for safety entails substantial ongoing commitments. Sustained resource allocation is required in performance monitoring, iterative software validation, and communication loops with clinicians and the call center for tech support [56, 62–64, 76]. Liability risks remain unresolved for predictive AI models, particularly given the lack of harmonized HTA methodologies for digital prevention [77, 78]. Moreover, payers remain cautious unless KPIs, such as engagement and clinical outcomes (e.g., HbA1c or weight reduction), demonstrate both health impact and clear cost savings.

In sum, accountability mechanisms in Switzerland can enhance venture credibility, but they also impose high evidentiary and compliance burdens. This dual role makes accountability a potential enabler of the B2B transition.

### (RQ2) What financial thresholds must be reached to achieve breakeven and investment viability during the B2C phase?

#### Breakeven probability analysis

**Fig. 5:**
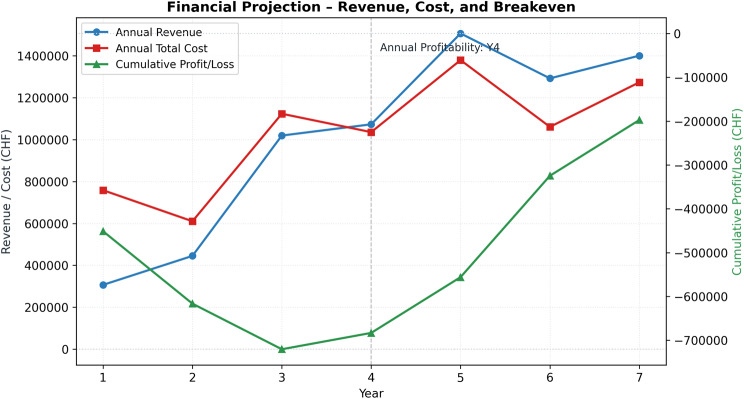
Financial base-case projection and breakeven analysis for a Swiss B2C diabetes screening venture

**Fig. 6:**
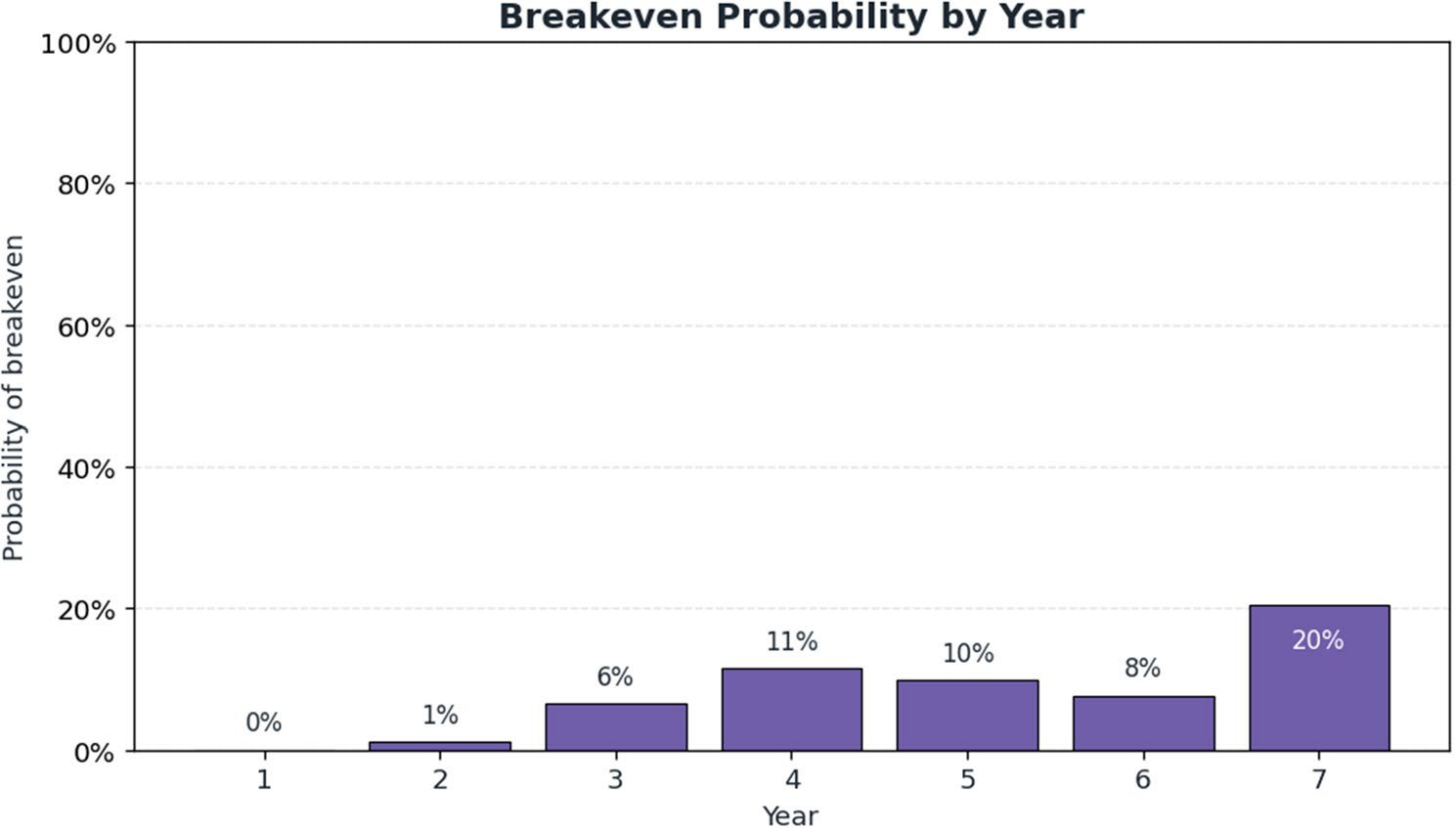
Breakeven probability distribution across financial projection (Years 1–7)

The venture generates its first positive annual cash flow in Year 4. The financial projection has not achieved cumulative break-even within Year 7, although cumulative cash flow reaches near positive at CHF −0.19 million, indicating that modest adoption trajectories can hinder a B2C digital diabetes screening in Switzerland. The market simulation in Fig. 5 shows that the B2C screening model can achieve a gradual, yet economically meaningful, scale under conservative adoption assumptions. Active users increase from 639 in Year 1 to nearly 3000 by Year 7, yielding a corresponding rise in annual revenue from CHF 0.31 million to CHF 1.39 million. The early years are characterized by high fixed and acquisition-related expenditures, resulting in negative cash flow through Year 3. The breakeven probability analysis in Fig. 6 indicates a 57% likelihood that cumulative cash flow becomes positive at any point within the seven-year horizon, with the highest annual probability of 20% in Year 7. Conversely, there remains a 43% chance that breakeven will never be achieved, reflecting substantial financial uncertainty despite the positive trajectory observed after Year 4.

#### Willingness-to-pay estimate

Collectively, sensitivity analyses results suggest that profitability is most plausible when three conditions align: pricing at or above CHF 40, CAC below CHF 200, and screening participation of at least 10%.

**Fig. 7:**
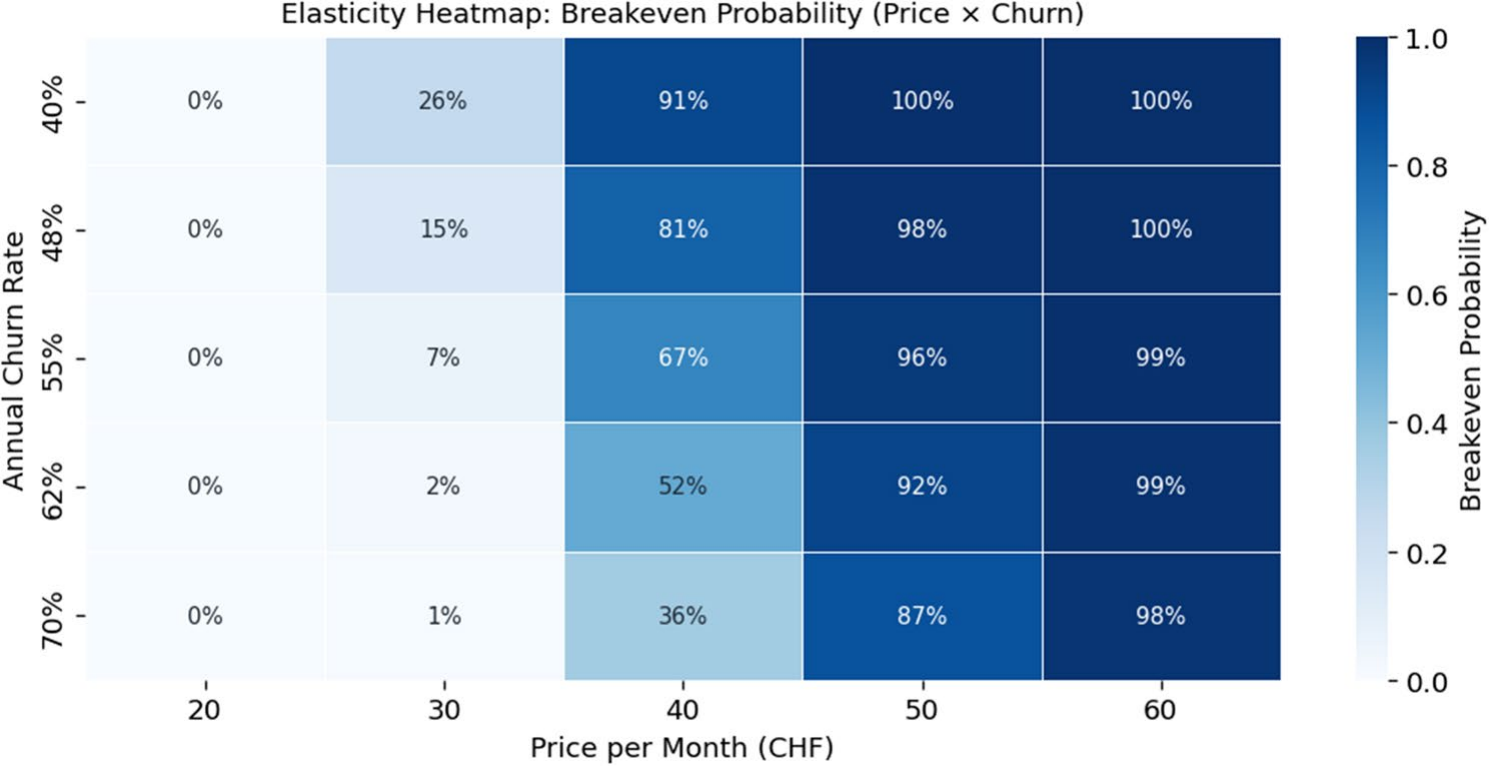
Elasticity heatmap of breakeven probability by price and churn rate

**Fig. 8:**
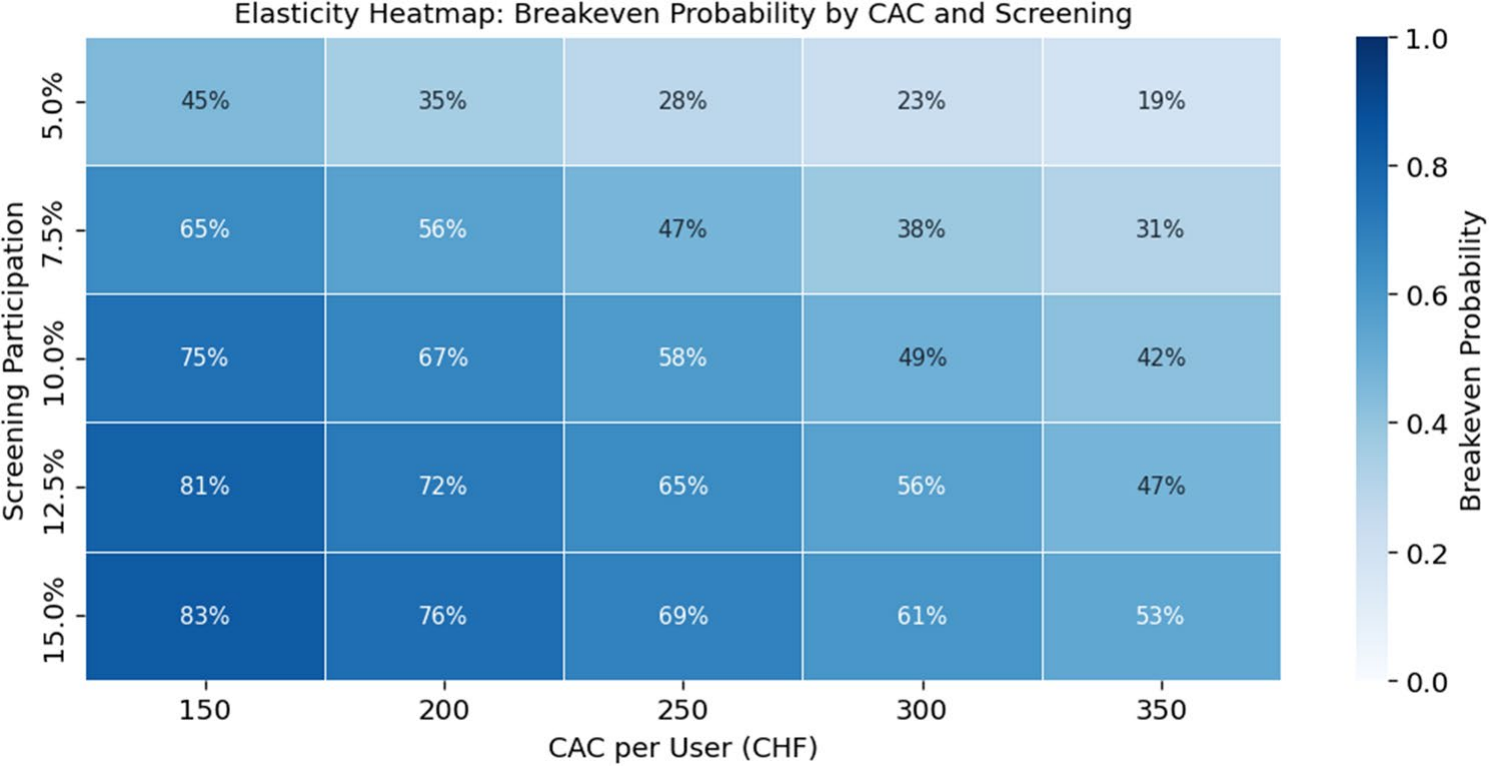
Elasticity heatmap of breakeven probability by CAC and screening participation

The sensitivity analyses in Figs. 7 and 8 show that the CHF 40 subscription is identified as a realistic price point given churn rate can be kept below 40%. At a lower price of CHF 20, revenues grow too slowly to offset acquisition, personnel, and infrastructure costs, yielding persistent early deficits and preventing cumulative breakeven. Although the base-case CHF 40 price achieves positive annual cash flow from Year 4 in Fig. 5, elasticity analyses indicate that CHF 20 fails to reach break-even under any churn scenario and yields consistently negative NPVs. By contrast, prices in the CHF 40–60 range align with the observed revenue scale-up and produce positive NPVs with notable breakeven probabilities (20%).

Elasticity analyses further delineate the conditions required for viability. Prices below CHF 30 generate uniformly negative NPVs across churn levels, whereas prices in the CHF 40–60 range produce positive NPVs in Fig. 7 that improve as churn decreases, confirming monthly price as the primary financial lever. The CAC-participation analysis shows that high CAC values ( > CHF 300) keep NPVs negative regardless of participation, while low CAC (CHF 150–200) combined with screening participation of ≥10% yields at least a 67% chance of achieving breakeven. Breakeven probabilities follow the gradient, increasing from 17% at 5% participation and CAC 350 to above 80% at 15% participation with CAC 150 in Fig. 8.

#### Valuation analysis

**Fig. 9:**
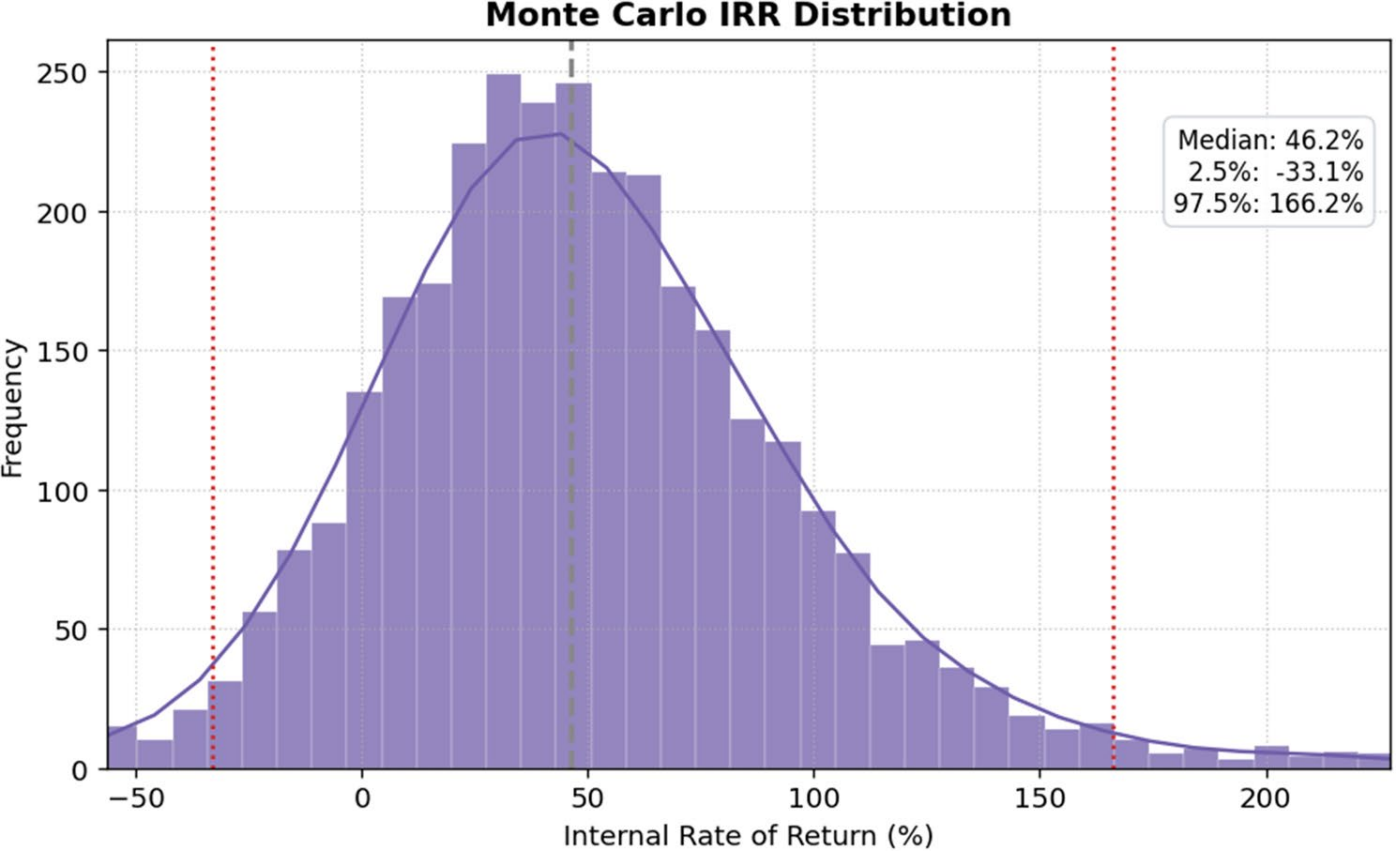
Monte Carlo internal rate of return (IRR) distribution

**Fig. 10:**
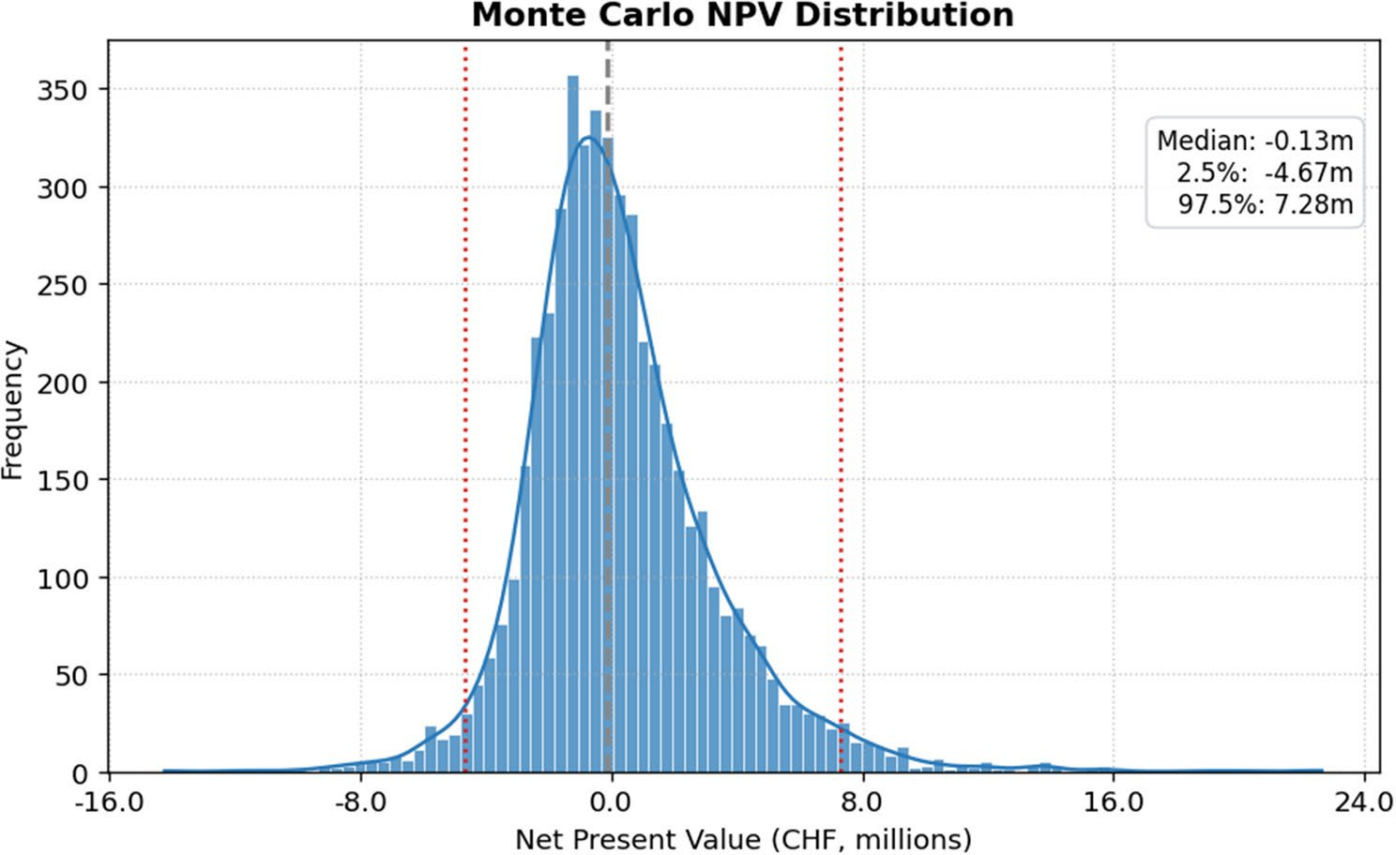
Monte Carlo net present value (NPV) distribution

A seven-year discounted cash flow (DCF) model was developed to value the B2C business case, using funnel-derived market projections and the bottom-up cost assumptions presented in Table 2. The financial metrics indicate a marginal probability of achieving profitability (57%), with the median IRR (46.16%) in Fig. 9 exceeding the typical hurdle rate of 20%. However, the median NPV is marginally negative (CHF −127,147) in Fig. 10, suggesting that while the rate of return is high, the overall value creation in the median scenario does not exceed the cost of capital over the project lifetime. The wide separation between the 2.5th and 97.5th percentiles for NPV and IRR reflects substantial uncertainty and right-skewed upside potential; the combination of high IRR with a slightly negative median NPV indicates limited absolute scale rather than model inefficiency, with high percentage returns arising from early cash inflows on a small capital base.

**Fig. 11:**
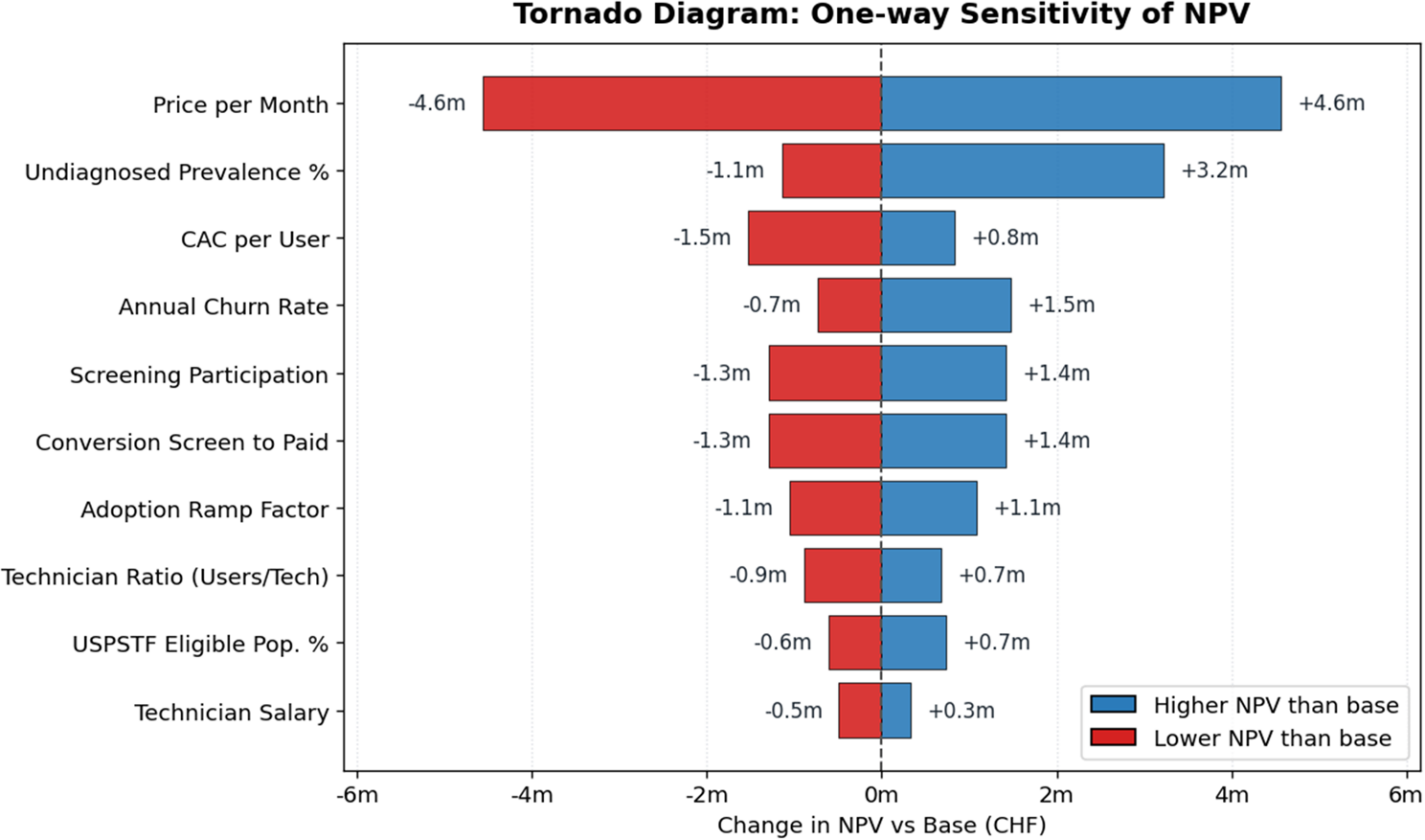
Tornado diagram of one-way sensitivity analysis of NPV

When assessed against the a priori viability criteria, the venture does not meet the requirement of achieving a ≥ 50% breakeven probability by Year 5, nor the criterion of positive NPV under several median and favorable scenarios. Yet it consistently approaches the IRR ≥20% threshold under stronger adoption conditions. One-way sensitivity analysis in Fig. 11 indicates that the monthly price is by far the dominant financial driver, with the greatest potential to shift outcomes from sustained losses to profitability.

### (RQ3) how is the implementation sustainability of this B2C model in Switzerland, particularly as it seeks to bridge towards B2B integration?

This analysis synthesizes findings from RQ1 (system alignment) and RQ2 (financial modeling) to determine whether a B2C venture could remain viable and serve as a bridge toward B2B integration.

#### Revenue potential

The revenue trajectory in the model grows steadily as the active user base expands to 2917 by Year 7, increasing annual revenue to CHF 1.39 million (Table 2). This growth, while gradual, is sufficient to offset rising operating costs over time, enabling the venture to achieve its first positive annual cash flow in Year 4, indicating that the revenue generated at scale can sustain both personnel and infrastructure costs. However, the results also show that early revenues remain substantially below total costs, and the model reaches financial viability only once user volumes are large enough for recurring subscription income to outpace fixed expenditures. Overall, revenue potential is positive but contingent on sustained growth in active users, with profitability emerging only in later years; however, while the B2C model is operationally feasible, standalone sustainability remains unlikely under current policy and reimbursement conditions due to persistent financing and regulatory constraints. Therefore, long-term sustainability might depend on strategic partnerships with payers and other existing health check-up providers. Integration into insurer wellness ecosystems, such as CSS’s Well, SWICA’s BENEVITA, or Helsana+, could enable white-label deployment, increasing achievable revenue potential.

#### Cost efficiency

The cost structure exhibits a substantial upfront investment, with annual expenses totaling CHF 0.76 million in Year 1, rising to CHF 1.27 million by Year 7 (Table 2). Personnel, customer acquisition, and backend services constitute the largest cost components and scale proportionally with user growth. Despite increasing absolute expenses, cost efficiency improves over time: as active users expand, the cost per active user decreases substantially, reflecting economies of scale. In particular, annual cash flow turns positive with 2235 active users in Year 4 and remains positive thereafter, indicating that the cost structure becomes sustainable once user volumes are sufficiently high. Overall, the results suggest that operational viability is achievable under moderate growth, with long-term sustainability dependent on maintaining cost discipline.

#### Managerial scalability

Managerial scalability is supported by a lean technician-to-user and manager-to-technician ratios, with staffing projected to grow from 1 to 5 full-time technicians, overseen by only one manager. This structure enables digital-first growth without the high personnel costs typically associated with data-intensive health ventures. The staffing profile in the results table assumes a scalable managerial structure, with technician salary costs rising from CHF 95,000 in Year 1 to CHF 475,000 in Year 7, while manager salary costs remain constant. This pattern suggests that technician capacity must scale with user growth, whereas managerial oversight is kept minimal, reflecting a lean administration. However, potential friction points include multilingual onboarding, technology integration, and customer support workflows, which can increase overhead if not managed effectively. Sustaining efficiency will require self-service infrastructure, scripted onboarding, and interoperable operating systems to minimize manual intervention. Overall, the lean staffing model is deemed crucial as a pathway for B2C growth, but long-term scalability depends on continuous investment in automation and customer operations tooling.

#### Technology adaptability

Technological adaptability benefits from Switzerland’s high digital penetration and strong familiarity with health apps, providing a solid foundation for user engagement. However, fragmented EHR systems across cantons, the absence of a unified patient identifier, and evolving SaMD regulatory requirements restrict seamless integration with clinical infrastructure. Sustained retention depends on real-time biometric and behavioral feedback loops, which require continuous system reliability and validation. To remain future-relevant, the venture must maintain a modular data pipeline with transparent validation and portability standards, ensuring compatibility across evolving digital biomarkers. Integration with commercial wearables and home diagnostic kits offers scope for service expansion, but long-term sustainability will hinge on meeting strict interoperability and compliance standards.

In summary, sustainability in the B2C phase is achievable through declining unit costs, lean staffing, and adaptable technology; however, each dimension is contingent upon overcoming compliance and interoperability barriers.

## Discussion

This study assessed the system fit and financial viability of a B2C digital diabetes screening venture in Switzerland, applying Herzlinger’s Innovating in Healthcare framework alongside pro forma modeling. Unlike retrospective models like Donabedian’s or payer-focused HTA, Herzlinger’s framework suits consumer-facing, pre-reimbursement innovations, where system alignment must be evaluated under uncertainty rather than established outcomes.

The results confirm prior findings that Switzerland offers strong consumer readiness and technological capacity for digital prevention [30, 66], while cost structures benefit from economies of scale once fixed investments are recovered [79]. In line with earlier work on digital health ventures [80], our analysis demonstrates that lean operating models can achieve efficiency gains over time. The novel contribution lies in quantifying that breakeven probability. Although the base-case scenario yields positive annual cash flow by Year 4, cumulative break-even is not achieved by Year 7 (CHF −0.19 million), and the venture fails the predefined viability criteria. This divergence illustrates that early-stage B2C prevention models may exhibit attractive return profiles while still failing to generate sufficient absolute value to justify standalone investment under conservative assumptions. Sensitivity analyses, however, demonstrate that financial sustainability, with an internal rate of return approaching or exceeding 20%, remains attainable under moderate consumer uptake alone when monthly pricing reaches at least CHF 40, customer acquisition costs remain below CHF 200, and screening participation is at least 10%, with price identified as the dominant driver. These findings challenge the prevailing assumption that early public-sector endorsement or reimbursement is essential for the viability of lean digital diabetes screening models in Switzerland.

Our key methodological contribution extends prior efforts [35] by systematically translating Herzlinger’s qualitative Six-Factor Framework [43] into a fully quantified neoclassical financial model using NPV decomposition and discounted cash flows, thereby converting structural and regulatory determinants into explicit economic consequences and adoption thresholds. Consistent with earlier critiques of Swiss healthcare governance [81, 82], we confirm that fragmented EHR systems, absence of reimbursement, and regulatory inertia constitute major systemic barriers. Unlike previous qualitative observations [59], we quantified their impact on consumer conversion, breakeven timing, and valuation, thus translating policy and system-fit constraints into measurable financial risks for digital prevention ventures.

As detailed in the six-factor assessment, the B2C model must comply with revFADP, GDPR, and MDR/MedDO requirements [32, 57] for Software as a Medical Device, necessitating robust consent management, privacy-by-design principles, encrypted hosting, processor agreements, and a comprehensive Data Protection Impact Assessment that addresses risks including unauthorized access, algorithmic bias, and misclassification. Although achievable, these compliance measures generate costs that contribute to early negative cash flows, delay breakeven, and increase uncertainty regarding timelines and evidence requirements for AI-based screening tools. Overall, the findings underscore that system-level misalignment, rather than technical feasibility, constitutes the primary bottleneck and that long-term viability will depend on enhanced structural support via payer partnerships, platform integration, or hybrid B2B reimbursement models.

These results position B2C screening not as a sustainable endpoint but as a transitional, evidence-building strategy. It creates option value by reducing uncertainty for subsequent B2B or B2B2C integration, rather than in immediate profit maximization. Long-term financial viability will ultimately require integration into B2B reimbursement pathways, such as insurer-sponsored wellness ecosystems (e.g., CSS’s active365, Helsana+, or SWICA’s BENEVITA). Although managerial scalability and regulatory accountability mechanisms establish a credible operational foundation, unresolved liability risks for predictive AI and the absence of established HTA methodologies for digital prevention constitute persistent barriers. Addressing these gaps will necessitate policy reforms to enable HTA frameworks and outcome-based reimbursement models. In the interim, alternative financing instruments, including Innosuisse innovation grants and Health Impact Bonds, could bridge the validation period and sustain ventures until payer integration becomes feasible.

### Limitations

This study has several limitations. First, the system-fit evaluation relied on secondary data and consensus-based scoring, which may introduce subjectivity. Second, the financial model used indirect estimates of the size of the market based on epidemiological data, pricing, and adoption from public and market data sources. Although assumptions were triangulated across government statistics, literature, and industry reports, they remain unvalidated proxies for consumer behavior. Third, willingness-to-pay was inferred from analogous preventive services rather than primary survey or experimental evidence, limiting external validity.

Future research should validate these assumptions through real-world pilots measuring actual adoption, conversion, and willingness-to-pay. A comprehensive HTA should be conducted to consider the perspectives of payers and providers, including cost-effectiveness analyses, sensitivity analyses, and budget impact studies. Finally, while this model assumed equity-only financing, future implementation may involve hybrid strategies (e.g., Innosuisse grants, early equity, or conditional reimbursement pilots), which could materially affect the breakeven requirement and valuation.

## Conclusions

A B2C digital diabetes screening model in Switzerland achieves only partial system alignment. Although breakeven appears feasible under favorable assumptions of price and customer acquisition cost, financial viability remains highly sensitive to willingness-to-pay and churn dynamics. The contribution of this work lies in quantifying, for the first time in the Swiss context, the thresholds at which a consumer-facing prevention model may become investable. However, its sustainability as a standalone model is limited. Strategic partnerships, alternative financing mechanisms, and transition to statutory reimbursement pathways will be essential to secure long-term integration into the Swiss healthcare system.

## Electronic supplementary material

Below is the link to the electronic supplementary material.

Supplementary Material 1

## Data Availability

All data generated or analyzed during this study are included in this published article. Additional source codes and details on financial modeling are available at DOI: 10.17605/OSF.IO/VH2FR.

## Abbreviations

ADA: American Diabetes Association
B2B: Business-to-Business
B2C: Business-to-Consumer
CAC: Customer Acquisition Cost
CE: Conformité Européenne
CHF: Swiss Francs
DCF: Discounted Cash Flow
DiGA: Digitale Gesundheitsanwendungen
DHTs: Digital Health Technologies
DPP: Diabetes Prevention Program
EAE: Efficacy, Appropriateness, Efficiency
EHR: Electronic Health Record
FSO: Federal Statistical Office
GDP: Gross Domestic Product
HTA: Health Technology Assessment
IRR: Internal Rate of Return
KPI: Key Performance Indicator
MDR: Medical Device Regulation (EU)
NCDs: Noncommunicable Diseases
NPV: Net Present Value
revFADP: revised Federal Act on Data Protection (Switzerland)
ROI: Return on Investment
SaMD: Software as a Medical Device
T2D: Type 2 Diabetes
USPSTF: United States Preventive Services Task Force
