# Supplementary material for: Early viability assessment of a Business-to-Consumer (B2C) model for digital diabetes screening in Switzerland
Source: BMC Health Serv Res. 2026 Jan 28;26:283. doi: 10.1186/s12913-026-14075-3 (PMC12924479; doi:10.1186/s12913-026-14075-3)
Supplement: Supplementary file 1 — Supplementary Material 1 [file 12913_2026_14075_MOESM1_ESM.docx]

**Appendix A**

This appendix presents evidence sources, all variable definitions, and mathematical equations used in the financial, diagnostic, and system-fit models.

**Table A1.** Evidence sources and extracted parameters across the six parameter groups

| Parameter Group | Purpose | Data Sources | Key Extracted Data |
| --- | --- | --- | --- |
| Epidemiology & Screening Eligibility | Define addressable population, undiagnosed rates, screening eligibility | IDF Diabetes Atlas 2025; United States Preventive Services Task Force 2024 | IFG 10-12%; undiagnosed diabetes up to 30%; up to 80% undetected in older adults |
| Clinical Effectiveness & Diagnostic Performance | Calibrate sensitivity, specificity, diagnostic accuracy | American Diabetes Association; Swiss Medical Weekly | ADA sensitivity 70-99%; HbA1c/FPG up to 50%; OGTT specificity 95-99%; voice-AI AUC up to 0.7 |
| Adoption, Adherence & Funnel Economics | Model awareness, conversion, retention | DigitalSwitzerland Survey | 90% smartphone ownership; 51% biomarker tracking |
| WTP, Prices & Consumer Behaviour | Set realistic B2C pricing ranges | Swiss HbA1c home-test prices; Swiss telemedicine price lists | HbA1c CHF 16; Home test kits CHF 70-75; CHF 20 teleconsultation fee |
| Cost & Comparator Pathways | Comparator costs for GP/pharmacy and interventions | Medics Lab Price List; Swiss dietitian/digital coaching costs | OGTT CHF 30-40 per test; counseling CHF 40 per 15min; coaching CHF 40-60 per month |
| Regulatory, Privacy & Compliance Costs | Quantify MDR, revFADP, IvDO, HTA, DiGA requirements | revFADP 2023; EU MDR 2017/745 | CE-MDR CHF10,000-30,000; revFADP CHF 15,000-40,000 per annum |

**Table A2.** Summary of variables and parameter descriptions

| Variable Symbol | Description |
| --- | --- |
| Market Sizing Inputs | |
| $P_{adult}$ | Adult population size |
| $P_{eligible}$ | Percentage of the adult population eligible for the service |
| $P_{IFG}, P_{undiag}$ | Percentages of the eligible population at risk (Impaired Fasting Glucose, Undiagnosed) |
| $P_{screen}$ | Long-run screening participation percentage |
| $P_{conv}$ | Conversion rate from screening participation to paid subscriber |
| Time-Dependent Inputs (Varies by Year $t$) | |
| $\alpha(t)$ | Adoption factor for year t ($\alpha(t)$<1) |
| $P_{month}(t)$ | Price per month per user |
| $\text{CAC}_{user}(t)$ | Cost to acquire one new user |
| $R_{tech}(t)$ | Active users supported per technician (Technician Ratio) |
| $R_{mgr}(t)$ | Technicians supported per manager (Manager Ratio) |
| $S_{tech}(t)$, $S_{mgr}(t)$ | Annual salary for a technician and a manager |
| $C_{call,user}(t)$ | Annual call center cost per active user |
| $C_{backend,user}(t)$ | Annual backend service cost per active user |
| $C_{rent,worker}(t)$ | Annual office rent cost per worker |
| $C_{supplies,worker}(t)$ | Annual office supplies cost per worker |
| $C_{dev}(t)$, $C_{mdr}(t)$, $C_{fadp}(t)$ | Year-dependent fixed costs (App Dev, CE MDR, FADP Compliance) |
| Calculated Metrics (Outputs) | |
| $N_{pop}$ | Number of eligible individuals |
| $N_{risk}$ | Total number of individuals at risk |
| $S_{max}$ | Long-run maximum number of subscribers |
| $N_{sub}(t)$ | New subscribers in year t |
| $U(t)$ | Active users at the end of year t |
| $R(t)$ | Annual Revenue in year t |
| $T(t)$, $M(t)$ | Number of technicians and managers required |
| $C_{\text{cost}}(t)$ | Various operational costs (CAC, salaries, rent, etc.) |
| $C_{total}(t)$ | Total Operating Costs in year t |
| $\text{CF}(t)$ | Cash Flow (Profit / Loss) in year t |
| $S_{TV}$ | Terminal Value Multiple (applied to last year) |

**Table A3.** Summary of mathematical formulations used to model pro forma Profit & Loss

| Calculation Step | Parameter | Mathematical Formulation |
| --- | --- | --- |
| Market Sizing | Eligible Population | $N_{pop}=P_{adult}\cdot P_{eligible}$ |
|  | Population at Risk | $N_{risk}=N_{pop}\cdot(P_{IFG}+P_{undiag})$ |
|  | Long-Run Max Subscribers | $S_{max}=N_{risk}\cdot P_{screen}\cdot P_{conv}$ |
| User Acquisition (Year t) | New Subscribers | $N_{sub}(t)=max([S_{max}\cdot\alpha(t)]-\sum_{i=1}^{t-1} N_{sub}(i),0)$ |
|  | Cumulative Subscribers | $\sum_{i=1}^{t} N_{sub}(i)=\sum_{i=1}^{t-1} N_{sub}(i)+N_{sub}(t)$ |
|  | Active Users | $U(t)=U(t-1)\cdot(1-\text{Churn})+N_{sub}(t)$ |
| Revenue Calculation | Annual Revenue | $R(t)=U(t)\cdot P_{month}(t)\cdot12$ |
| Personnel Sizing | Number of Technicians | $T(t)=\lceil U(t)/R_{tech}(t)\rceil$ |
|  | Number of Manager | $M(t)=\lceil T(t)/R_{mgr}(t)\rceil$ |
| Cost Calculation | CAC Cost | $\text{CAC}_{C}(t)=N_{sub}(t)\cdot\text{CAC}_{user}(t)$ |
|  | Technician Cost | $C_{tech}(t)=T(t)\cdot S_{tech}(t)$ |
|  | Manager Cost | $C_{mgr}(t)=M(t)\cdot S_{mgr}(t)$ |
|  | Call Center Cost | $C_{call}(t)=U(t)\cdot C_{call,user}(t)$ |
|  | Backend Service Cost | $C_{backend}(t)=U(t)\cdot C_{backend,user}(t)$ |
|  | Office Rent Cost | $C_{rent}(t)=(T(t)+M(t))\cdot C_{rent,worker}(t)$ |
|  | Office Supplies Cost | $C_{supplies}(t)=(T(t)+M(t))\cdot C_{supplies}(t)$ |
|  | Total Operating Costs | $C_{total}(t)=C_{tech}(t)+\cdots+C_{fadp}(t)$ |
| Cash Flow | Cash Flow (CF) | $\text{CF}(t)=R(t)-C_{total}(t)$ |
|  | Adjusted Final CF | $\text{CF}_{adj}(t_{last})=\text{CF}(t_{last})\cdot S_{TV}$ |

**Table A4.** A seven-year pro forma Profit & Loss statement

| **Year** | **1** | **2** | **3** | **4** | **5** | **6** | **7** |
| --- | --- | --- | --- | --- | --- | --- | --- |
| Active Users | 639.6 | 927.42 | 2122.94 | 2234.52 | 3137.54 | 2691.09 | 2916.59 |
| New Subscribers | 639.6 | 639.6 | 1705.6 | 1279.2 | 2132 | 1279.2 | 1705.6 |
| CAC Cost | 140712 | 140712 | 375232 | 281424 | 469040 | 281424 | 375232 |
| Technician Salary | 95000 | 190000 | 380000 | 380000 | 475000 | 380000 | 475000 |
| Manager Salary | 120000 | 120000 | 120000 | 120000 | 120000 | 120000 | 120000 |
| Call Center Cost | 12792 | 18548.4 | 42458.78 | 44690.45 | 62750.7 | 53821.82 | 58331.82 |
| Backend Service | 22386 | 32459.7 | 74302.87 | 78208.29 | 109813.73 | 94188.18 | 102080.68 |
| Office Rent Cost | 20000 | 30000 | 50000 | 50000 | 60000 | 50000 | 60000 |
| Office Supply Cost | 2400 | 3600 | 6000 | 6000 | 7200 | 6000 | 7200 |
| App Dev Cost | 300000 | 50000 | 50000 | 50000 | 50000 | 50000 | 50000 |
| CE MDR Cost | 20000 | 0 | 0 | 0 | 0 | 0 | 0 |
| FADP Compliance | 25000 | 25000 | 25000 | 25000 | 25000 | 25000 | 25000 |
| Annual Revenue | 307008 | 445161.6 | 1019010.72 | 1072570.82 | 1506016.87 | 1291723.59 | 1399963.62 |
| Total Costs | 758290 | 610320.1 | 1122993.65 | 1035322.74 | 1378804.43 | 1060433.99 | 1272844.5 |
| Profit / Loss | -451282 | -165158.5 | -103982.92 | 37248.08 | 127212.44 | 231289.6 | 127119.12 |
| Cumulative CF | -451282 | -616440.5 | -720423.42 | -683175.34 | -555962.9 | -324673.31 | -197554.19 |
